# Comparative analysis of the lipid profiles of bovine fast- and slow-type muscles

**DOI:** 10.1016/j.fochx.2025.102569

**Published:** 2025-05-20

**Authors:** Heling Li, Xiaofan Tan, Yize Weng, Lei Zhang, Xuehai Du, Dawei Bian, Yangzhi Liu, Songyang Shang, Peng Li, David M. Irwin, Shuyi Zhang, Bojiang Li

**Affiliations:** a College of Animal Science and Veterinary Medicine, Shenyang Agricultural University, Shenyang 110866, China; b Liaoning Agricultural Development Service Center, Shenyang 110032, China; c Wellhope Foods Company Limited, Shenyang 110164, China; d Department of Laboratory Medicine and Pathobiology, University of Toronto, Toronto, ON M5S 1A8, Canada

**Keywords:** DALs, Meat quality, Lipid molecules

## Abstract

Lipid molecules are an important component of meat and are the main source of flavor. However, at present the lipid molecules in meat with different muscle fiber types in cattle are not fully understood. We carried out lipid profiling of eight fast-type *longissimus dorsi* (LD) muscle and eight slow-type *psoas major* (PM) muscle samples from LYWC (Liaoyu white cattle). A total of 2032 lipid molecules were identified by our lipidomic analysis of these two muscle tissues, and 134 lipid species were identified as differentially abundant lipids (DALs). A correlation analysis showed the close relationship between the DALs identified in the LD and PM muscle. In addition, some DALs are closely associated with meat quality traits and muscle fiber types. This study identifies lipid molecules that differ between meats with different muscle fiber types and provides new insights into the role of lipids in beef quality improvement.

## Highlights

• A total of 2032 lipids were obtained from these LD and PM meat samples.
• The 134 differential lipids were identified between LD and PM meat.
• Some DALs affect the meat quality by influencing the flavor substance formation.

## 1 Introduction

Cattle are an important source of meat for human consumption. Beef offers many essential nutritional ingredients, such as proteins, amino acids, fats, minerals, and vitamins (Vahmani et al., 2020). With changes in the composition of the meats consumed in China and other places, the demand for beef has been increasing every year. Skeletal muscle represent the largest organ in cattle, and accounts for about 40 %–50 % of total body weight (Apponi, Corbett, & Pavlath, 2011). Skeletal muscle is a complex and variable tissue consisting of a large group of heterogeneous fibers, which typically make up 75–90 % of the muscle volume (Dos Santos et al., 2023; Lee, Joo, & Ryu, 2010). Skeletal muscle fibers are classified into four predominant types according to the predominant myosin heavy chain (MyHC) isoform: slow oxidative (MyHC I), fast-twitch oxidative glycolytic (MyHC IIA), fast glycolytic (MyHC IIB) and intermediate (MyHC IIX) (Tan et al., 2023). Slow oxidative muscle fibers contain higher levels of mitochondria and myoglobin and are mainly involved in oxidative metabolism, while fast glycolytic muscle fibers have lower levels of mitochondria and higher ATPase activity and glycolytic metabolism (Schiaffino & Reggiani, 2011). In our previous study, the number of type I muscle fibers in PM was shown to be significantly greater than in the LD by SDH staining, whereas the number of type II muscle fibers was significantly less than in the LD (Tan et al., 2025). Many previous studies have confirmed that muscle fiber type is critical to meat quality. Park et al. observed a negative correlation between relative type I fiber area and shear force whereas it was positively correlated with fat content (Park et al., 2024). Increasing the proportion of type I muscle fibers increases the pH and meat color *L* value, and reduces the cooking loss of meat (Li et al., 2022). Bai et al. showed that the abundance of type I muscle fibers was positively correlated with myosin content, antioxidant activity, intramuscular fat content, and saturated fatty acid in meat (Bai et al., 2022).

Lipid molecules have a crucial role in influencing the nutritional, flavor, sensory and other characteristics of meat (Prates, 2025). For example, lipid molecules are critical for the formation of flavor in meat products, which influence consumer acceptance through the sensory perception of meat. Recently, several studies used lipidomic techniques to identify the lipid expression profiles in meat and identified biomarker lipid molecules associated with their characteristics (Lam, Wang, Li, & Shui, 2021). Antonelo et al. comprehensively characterized the lipid profiles of high and normal pH beef, and uncovered that lipid molecules influence the final pH of meat through glycolytic and other energy metabolism pathways (Antonelo et al., 2022). Some investigators have identified and analyzed lipid molecules in the mitochondria from bovine longissimus lumborum (LL) muscle and psoas major (PM) meat (Zou et al., 2023). A recent study explored the effects of flaxseed oil cyclolinpeptides on lipid molecules during the degradation of high-fat meat in cattle using untargeted lipidomic techniques (Mueed et al., 2025). A lipidome-based identification of Allium mongolicum regel bulb powder supplementation showed that it was significantly associated with changes in the abundance of 199 lipid molecules in beef (Liu et al., 2024). Nevertheless, the identification of the lipid profiles in beef, especially muscles with differing muscle fiber types, has not been exhaustive.

Liaoyu white cattle (LYWC) is a breed produced through crossbreeding of Charolais cattle and Liaoning local yellow cattle, which has the advantages of fast growth, high meat quality and strong resistance. Our previous study found that fast-type *longissimus dorsi* (LD) and slow-type *psoas major* (PM) muscles of LYWC have significantly different meat qualities (Tan et al., 2025). In addition, their transcriptomic, proteomic and metabolomic profiles were comprehensively analyzed in a previous study, however, their lipid profiles are not known (Tan et al., 2025). This current study characterized the lipidomic profiles of LD and PM muscles, identified lipid substances that differ between them, and conducted a correlation analysis of the differential lipids. In addition, correlation analyses of DALs with meat quality traits and muscle fiber types were performed. Data from this study should help identify lipids that distinguish different muscle fiber types of meat and provide a basis for investigations to improve beef quality.

## 2 Materials and methods

### 2.1 Animals and sample collection

Eight Liaoyu white cattle (LYWC) used for this study were described in a previous study (Tan et al., 2025). These cattle were raised under the same diet and environment conditions and slaughtered in a commercial abattoir by electrocution and exsanguination based on standardized processes. *Longissimus dorsi* muscle (LD) between the 12th and 13th ribs and *psoas major* muscle (PM) were collected from the post-slaughter left side of carcasses. Muscles from each individual served as a biological repetition. Collected muscle samples were immediately frozen in liquid nitrogen, and then returned to the laboratory for storage at −80 °C. Experimental animals in the study were operated in accordance with the guide for the care and use of laboratory animals established by the Ministry of Agriculture of China. The Ethics Committee and the Experimental Animal Committee of Shenyang Agriculture University approved all animal experiments (permit number 202006032; March 30, 2020).

### 2.2 Extraction of lipids

Lipids from the muscle samples were extracted according to the MTBE method (Guedes et al., 2020). Briefly, appropriately 30 mg of sample was first spiked and then homogenized using homogenizer (MP Biomedicals, USA) with 200 μll water and 240 μll methanol. The homogenate was supplemented with 800 μll of MTBE and subjected to ultrasound with ultrasonic liquid processors (Scientz, China) for 20 min at 4 °C, and then kept at room temperature for 30 min. Subsequently, the solution was centrifuged at 14,000*g* for 15 min at 10 °C to yield an upper organic solvent layer, which was then dried by nitrogen and stored at −80 °C for later use.

### 2.3 LC-MS/MS analysis of the lipids

The lipid extract was re-dissolved with 200 μl 90 % isopropanol/acetonitrile with vortexing using vortex mixer (QiTe, China), with 90 μl of the re-dissolved solution then centrifuged using high-speed centrifuge (Eppendorf, Germany) at 14,000*g* at 10 °C for 15 min to obtain the supernatant. LC separation was performed using UHPLC Nexera LC-30A system (SHIMADZU, Japan) with a CSH™ C18 column (1.7 μm, 2.1 mm × 100 mm, Waters). The mobile phase A was 60 % acetonitrile and 40 % water with 0.1 % formic acid and 0.1 mM ammonium formate and mobile phase B was 10 % acetonitrile and 90 % isopropyl alcohol. The mobile phase was initially 40 % solvent B, and the flow rate was 300 μl/min. The gradient elution process was set as follows: 0–3.5 min, solvent B was kept at 40 %, 3.5–9 min, solvent B was linear from 40 % to 75 %; 9–15 min, solvent B was linear from 75 % to 99 %; 15–20 min, solvent B was kept at 40 %. Mass spectra was acquired by Q-Exactive Plus (Thermo Scientific, USA) in positive and negative mode. The ESI parameters were as follows: the source temperature was 300 °C, the capillary was 350 °C, the ion spray voltage was 3000 V, the RF level of the S-lens was 50 %, and the scanning range was *m*/*z* 200–1800. Ten fragmentation spectra (MS2, HCD) were taken for each complete scan. The resolutions of MS1 and MS2 were 70,000 and 17,500, respectively, at *M*/*Z* 200.

### 2.4 Data analysis

Peak signal recognition, peak extraction, and identification of lipid molecules were performed with LipidSearch software (Thermo Scientific™). The mass tolerance for precursors and fragments of this parameter were set to 5 ppm. Orthogonal partial least squares discriminant analysis (OPLS-DA) method was employed for principal component analysis of the lipid substances of the samples. In this study, OPLS-DA variable significance of projection (VIP > 1, *P* value <0.05) was used as a screening criterion for significant differences in lipid substances.

## 3 Results

### 3.1 Lipid profiling of longissimus dorsi (LD) and psoas major (PM) muscle in cattle

**Fig. 1:**
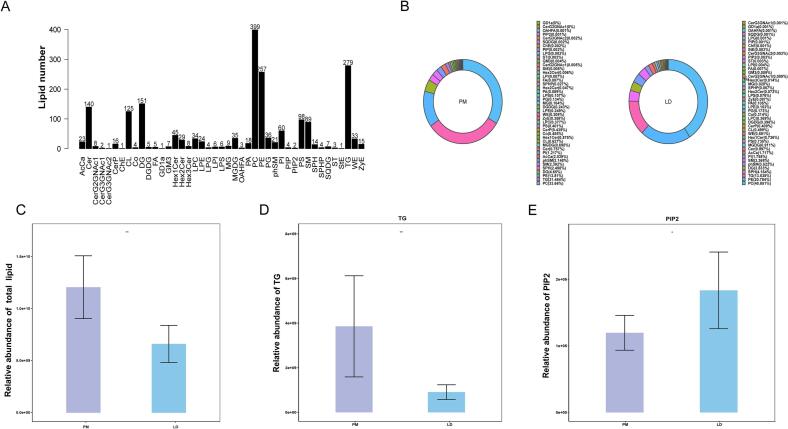
Classification of the lipids identified in LD and PM muscle. (A) Statistical chart of the lipid classes and species quantities in the muscle samples. (B) Proportions of the identified lipid class composition in PM and LD muscle. (C) Total lipid molecule content of LD and PM muscle. (D) TG molecule content of LD and PM muscle. (E) PIP2 molecule content of LD and PM muscle.

In this study, we analyzed the lipid profile of LD and PM muscle, based on eight samples for each muscle. A total of 2032 lipid compounds were identified by LC-MS/MS method from the muscle samples (**Table S1**), which were classified into 43 lipid subclasses (Fig. 1A). The greatest number of lipids were categorized as phosphatidyl-choline (PC; 399, 19.64 %), followed by triglyceride (TG; 279, 13.73 %) and phosphatidyl-ethanolamine (PE; 257, 12.65 %). The outcome of the compositional analysis of the lipid subclasses of LD and PM muscles is displayed in Fig. 1B. For LD muscle, the lipid subclasses PC, PE, TG, SPH, and DG accounted for 40.85 %, 20.78 %, 13.54 %, 4.16 %, and 3.53 %, respectively. In contrast, the lipid subclasses PC, PE, TG, SPH, and DG accounted for 33.66 %, 13.81 %, 31.48 %, 2.50 %, and 4.65 % in the PM muscle, respectively. The total abundance levels of the identified lipid molecules was further analyzed, which showed that the total lipid content in PM muscle was significantly higher than in LD muscle (Fig. 1C). In addition, the abundance of the lipid subclasses changed distinctly between the two muscles. For instance, TG content was markedly higher in PM muscle than in LD muscle (Fig. 1D). Nevertheless, PIP2 levels were significantly decreased in PM compared to LD (Fig. 1E).

### 3.2 Orthogonal partial least squares discriminant analysis (OPLS-DA) analysis

**Fig. 2:**
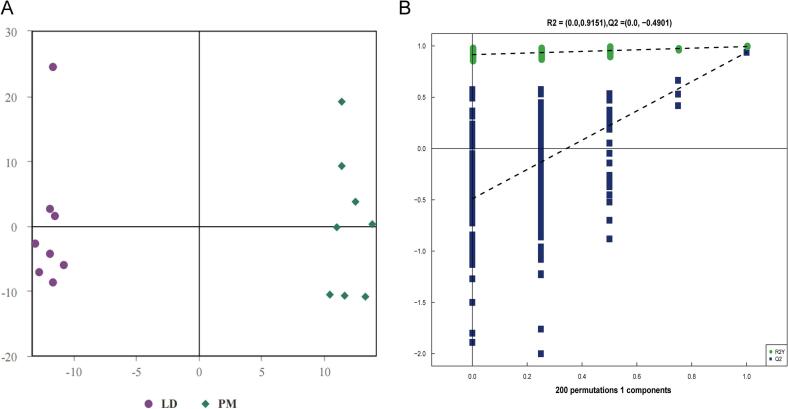
Results of the OPLS-DA model analysis of the lipidomics between LD and PM muscle. (A) OPLS-DA score plot and (B) permutation score plot obtained from the LD and PM muscle samples.

In this study, OPLS-DA was used to investigate the difference in the lipid profiles between the LD and PM muscle samples. The OPLS-DA result show that the lipidomic profiles could clearly separate the LD and PM samples, indicating that there were significant differences between them (Fig. 2A). The OPLS-DA model analysis obtained an R^2^Y of 0.994 and a Q^2^ of 0.938 (Fig. 2B), indicating that the OPLS-DA model is reliably stable. To determine whether overfitting occurred in this model, we performed a permutation test on the model, with the results showing that the intercepts of R^2^ and Q^2^ intercept are 0.915 and − 0.490 (Fig. 2B), respectively, indicating that this model is not overfitted. Taken together, the LD and PM muscles clearly separate in the OPLS-DA model.

### 3.3 Identification of differentially abundant lipids

**Fig. 3:**
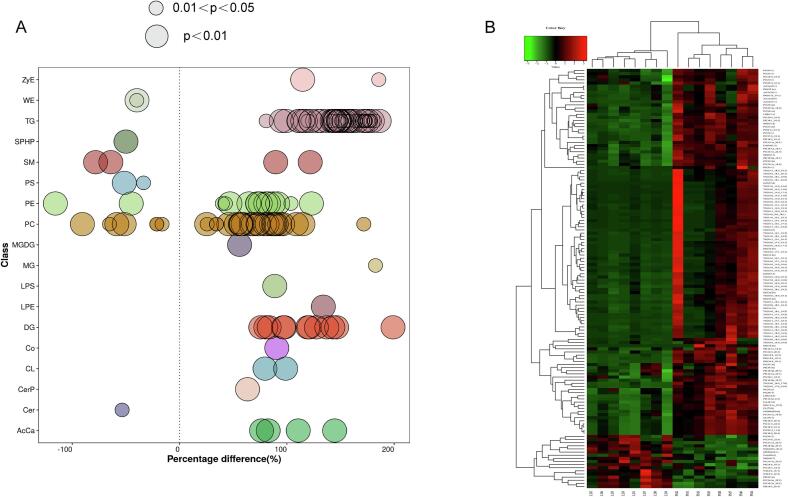
Identification of differentially abundant lipid (DAL) molecules between PM and LD muscles. (A) Bubble plot for the differentially abundant lipids in each lipid subclass. Bubbles represent lipids and its size represents significance. (B) Hierarchical cluster and heatmap depicting the detected DALs between LD and PM muscle samples. Eight replicates were examined for LD (LD1 to LD8) and PM (PM1 to PM8) muscles. Each block in the heatmap represents the abundance level of the DALs, with red and green representing relatively higher and lower abundance levels detected in the LD and PM muscle, respectively. (For interpretation of the references to colour in this figure legend, the reader is referred to the web version of this article.)

In the present study, a *P* value of <0.05 and a VIP value of >1 were used to identify differentially abundant lipids (DALs) between the LD and PM muscle samples. Between the muscle samples, 134 lipids were identified as DALs with 117 being up-regulated and 17 being down-regulated in PM muscle (**Table S2**). The DALs were categorized into 18 lipid subclasses including ZyE, WE, TG, SPHP, SM, PS, PE, PC, monogalactosyldiacylglycerol (MGDG), MG, LPS, LPE, DG, Co, CL, CerP, Cer, and acylcarnitine (AcCa) (Fig. 3A). Heatmap visualization and cluster analysis of the identified DALs (134 lipids) between the LD and PM muscle samples are depicted in Fig. 3B. It was found that the eight biological replicate samples for LD and PM muscles clearly clustered, indicating that there are different content patterns for these DALs in the two types of muscle.

### 3.4 Correlation analysis between DALs

**Fig. 4:**
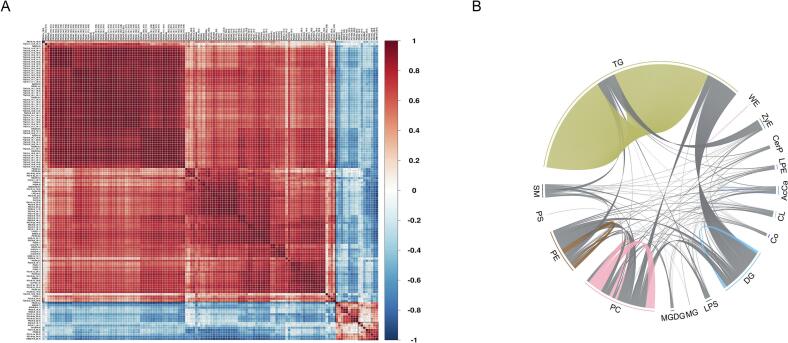
Correlation analysis of the DALs between the LD and PM samples. (A) Correlation clustering heatmap showing the correlation analysis results between DALs, with red and blue dots representing positive and negative correlations, respectively. (B) Chord diagrams illustrating lipid interactions with correlation coefficients greater than 0.8. The colored lines indicate correlations between lipid molecules within classes, and the grey lines indicate correlations between classes. (For interpretation of the references to colour in this figure legend, the reader is referred to the web version of this article.)

A correlation analysis was performed using the identified DALs between the LD and PM muscle samples, with the results being shown in Fig. 4A. The obtained results indicated that several DALs showed significant positive correlations, e.g., PC (24:2_11:2) and PE (18:0_20:4) (*r* = 0.9999), TG (15:0_18:1_18:2) and TG (16:1_16:1_18:2) (*r* = 0.9945), DG (33:0e) and TG (18:0_17:1_18:1) (*r* = 0.9908) and many others. In addition, TG (18:0_17:0_18:0), DG (18:0_18:2), PC (14:0_22:4) showed significant negative correlations with the molecules of PC (16:0e_22:5) (*r* = −0.7715), PE (18:0p_20:5) (*r* = −0.7714), PE (18:1_18:1) (*r* = −0.7695), respectively (**Table S3**). Associations with correlation coefficients greater than 0.8 and *p*-values less than 0.05 between DALs are displayed in Fig. 4B.

### 3.5 Correlation analysis for DALs with meat quality and muscle fiber types

**Fig. 5:**
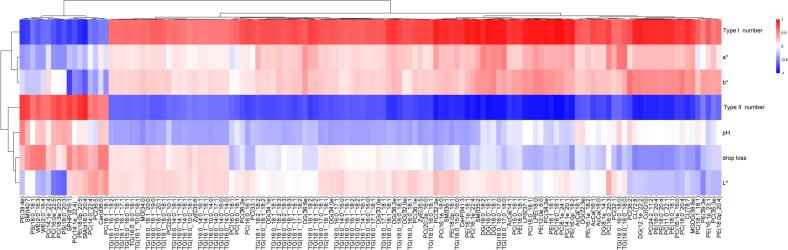
Correlation analysis of DALs with meat quality and muscle fiber type. The column indicates the meat quality and muscle fiber type parameters, and the row indicates the DALs. The correlation between them is shown by the different colors, and the red and blue represents the positive and negative correlation, respectively. (For interpretation of the references to colour in this figure legend, the reader is referred to the web version of this article.)

To evaluate the correlation between meat quality and muscle fiber types and DALs, we calculated correlation coefficients for DALs with meat quality traits including meat color (L*, a* and b*), pH, drip loss and muscle fiber proportions from the same samples in a previous study (Tan et al., 2025). Correlation analyses of meat quality and muscle fiber types with DALs were then performed on both samples (Fig. 5**)**. For example, there were a positive correlation between type I fiber number and PC (18:1_18:1), a* and TG (18:0_18:0_18:0). A negative correlation was observed between type II fiber number and PE (18:1_18:1), b* and PS (18:0_18:1).

## 4 Discussion

Lipid substances play important roles in meat quality, including texture, juiciness, flavor, and sensory characteristics. Many prior works have identified a vast number of lipid molecules in beef by lipidomic techniques. Recent research identified 472 lipid molecules from bovine longissimus lumborum (LL) muscle (Krauskopf et al., 2025). Similarly, other investigators identified 1032 lipid molecules and classified into 3 lipid classes and 8 subclasses in irradiated marble beef based on MS techniques (Zhang et al., 2023). In our study, a total of 2032 lipid molecules, categorized into 43 lipid classes, were detected from meat samples from 8 LD and 8 PM muscles, which is a higher number than found in previous studies. This also suggests that the lipid profile in beef is not entirely understood. The lipid substances identified here provide the basis for future work on the identification of meat quality related biomarkers. The results of lipidomic analyses indicate that PC, TG, and PE are the three primary lipid subclasses in beef muscle examined here, which is in line with the results of Zhang et al. for pork and Liu et al. for beef (Liu, Ma, et al., 2024; Zhang, Liu, Yang, Zhang, & Wang, 2022). These observations suggest that the three lipid subclasses TGs, PCs, and PEs are highly abundant in meat from different species. An interesting outcome of our analysis was that PM muscle has a higher total lipid abundance than LD muscle meat. This phenomenon was also observed in the lipid profiles of the mitochondria from both of these muscles (Zou et al., 2023).

A previous study has suggested that phosphatidylcholine (PC) and phosphatidylethanolamine (PE) have essential roles in the production of flavor substances through lipid oxidation (Zhang et al., 2025). Zhang et al. observed higher levels of PC using lipodomic techniques in Jianhe White Xiang pigs, which have superior meat quality, than in Large White pigs (Zhang et al., 2022). Liu et al. found that some PC and PE molecules were associated with the formation of major aroma compounds in roast mutton (Liu et al., 2022). Moreover, phospholipids, including PC and PE, are important lipid components of roasted chicken skin where they contribute to the production of aromatic compounds (Nie et al., 2024). In our results, the levels of the lipid subclasses PC and PE were significantly higher in the slow muscle PM than in fast muscle LD, suggesting that PC and PE may contribute to the meat quality of PM meat by affecting its flavor. In addition, a total of 33 lipids in the PC subclass had significantly different abundances between LD and PM meat, including PC (18:1_18:1). Liu et al. obtained a significant positive correlation between PC (18:1_18:1) and many flavor compounds in their correlation analysis (Liu et al., 2024). A differential lipid molecule, PE (18:1_18:1), was significantly negatively correlated with the flavor compound butanoic acid (Liu, Gao, et al., 2024). In the present study, we found a positive correlation between PC (18:1_18:1), PE (18:1_18:1) and meat qualities such as type I fiber number, a*, b*. These results suggest that these key lipid molecules influence the flavor of PM and LD muscle through the oxidative processes.

TGs play an important role as the main form of lipid storage and energy production as well as serving as a transporter of fatty acids (FAs) in the organism (Wang et al., 2022; Zhou, Zhao, Bindler, & Marchioni, 2014). In our study, we found that the total level of TGs was higher in PM muscle than in LD muscle. TG are thought to be the major lipid constituents of pork and have been shown to be a major factor influencing IMF levels and meat taste (Chen et al., 2020; Li et al., 2020). Wang et al. demonstrated that TG content was significantly higher in Laiwu pigs with high intramuscular fat than in those with low intramuscular fat (Wang et al., 2023). These data are in agreement with the observation that PM muscle has higher intramuscular fat content and flavor than LD muscle, which contributes to PM muscle having a superior meat quality. Some studies have obtained similar results, for example, Liu et al. found that TG (16:0_18:1_18:1) and TG (18:0_18:0_18:1) are the main lipids associated with mutton aroma. Liu et al. found that TG (16:0_18:1_18:1) are potentially key lipids producing odorants in roasted pork (Liu et al., 2024). Zhou et al. found that TG (16:0_18:1_18:1), TG (16:0_18:1_18:2), and TG (16:0_16:1_18:1) play important roles in the generation of key aroma compounds in beef (Zhou et al., 2024). Liu et al. demonstrated that TG (18:0_18:0_18:1), TG (16:0_14:0_18:1), TG (18:0_17:0_18:1), TG (18:0_16:0_18:1), TG (16:0_16:0_18:1), TG (15:0_16:0_18:1), and the volatile compound ethyl acetate have a significant positive correlation in their abundance, with ethyl acetate contributing to the flavor of meat (Liu, Ma, et al., 2024). These suggest that they play a significant role in producing aromatic compounds among the different kinds of muscle fiber.

Cardiolipin (CL), which is mainly phospholipid, plays a key role in the formation and maintenance of protein-protein interactions, as well the stabilization of the mitochondrial respiratory chain (Li et al., 2019). A previous study found that Laiwu pigs, which have a greater proportion of slow-type muscle fibers, have a higher content of CLs than Yorkshire pigs (Hou et al., 2023). The lipid molecule CL affects meat quality by influencing cellular energy metabolism, apoptosis, and the biological functions of mitochondrial cytochrome *c* (Zou et al., 2023). In our study, we found that the levels of CLs including CL (78:7) and CL (72:8) were higher in PM muscle than in LD muscle. Furthermore, correlation analyses also showed a positive correlation between CL (72:8) and type I fiber number. These results suggest that CLs contribute to the function of mitochondria and lead to better meat quality in slow-type PM muscle.

A LPE lipid molecule, LPE (18:0), was distinctly higher in PM meat than in LD meat. Previous studies have reported that LPE is primarily involved in glycerophospholipid metabolism and that the LPE (18:0) levels are increased by cold storage of chicken (Lv et al., 2023). Similarly, Jia et al. found that its level increased during the cold chain storage of lamb (Jia et al., 2021). In the present study, our correlation analyses also showed a positive correlation of LPE (18:0) with the number of type I fiber, and a negative correlation with the number of type II fiber. These observations suggest that LPE lipid molecules may influence post-slaughter meat quality of beef with different muscle fiber types.

## 5 Conclusion

The lipid profiles of LD and PM muscles in LYWC cattle were compared and a total of 2032 lipids, categorized into 43 subclasses, were identified from 8 samples of each of these two types of muscles. Of these, 134 showed significant different abundances (DALs, differentially abundant lipids) between LD and PM meat. These differentially abundant lipid molecules, including those in the lipid subclasses PC, PE, and TG, potentially affect the meat quality of both types of meat by influencing flavor substance formation processes. Our study identifies some lipid markers that have different abundances between the different muscle fiber meats and provides new insights to understand how lipid molecules affect beef quality. The mechanism of differential lipid substance effects on muscle fibers will be further explored in future studies.

## CRediT authorship contribution statement

**Heling Li:** Writing – original draft, Investigation, Formal analysis. **Xiaofan Tan:** Writing – original draft, Data curation, Conceptualization. **Yize Weng:** Project administration, Conceptualization. **Lei Zhang:** Funding acquisition, Formal analysis. **Xuehai Du:** Project administration. **Dawei Bian:** Supervision. **Yangzhi Liu:** Supervision, Data curation. **Songyang Shang:** Conceptualization. **Peng Li:** Funding acquisition, Conceptualization. **David M. Irwin:** Writing – review & editing. **Shuyi Zhang:** Project administration, Conceptualization. **Bojiang Li:** Writing – review & editing, Funding acquisition.

## Declaration of competing interest

The authors declare that they have no known competing financial interests or personal relationships that could have appeared to influence the work reported in this paper.

## Data Availability

Data will be made available on request.

## Appendix A Supplementary data

Supplementary material 1 Table S1 The lipid molecules identified in this study. Supplementary material 1

Supplementary material 2 Table S2 List of differentially abundant lipids. Supplementary material 2

Supplementary material 3 Table S3 Correlation analysis between DALs. Supplementary material 3
